# Use of the Free Endometriosis Risk Advisor App as a Non-Invasive Screening Test for Endometriosis in Patients with Chronic Pelvic Pain and/or Unexplained Infertility

**DOI:** 10.3390/jcm12165234

**Published:** 2023-08-11

**Authors:** Camran Nezhat, Ellie Armani, Hsuan-Chih Carolina Chen, Zahra Najmi, Steven R. Lindheim, Ceana Nezhat

**Affiliations:** 1 Camran Nezhat Institute, Center for Special Minimally Invasive and Robotic Surgery, Woodside, CA 94061, USA; 2 Stanford University Medical Center, Palo Alto, CA 94305, USA; 3 University of California San Francisco, San Francisco, CA 94143, USA; 4 Department of Obstetrics and Gynecology, Boonshoft School of Medicine, Wright State University, Dayton, OH 45324, USA; 5 Department of Obstetrics and Gynecology, University of Central Florida, Orlando, FL 32827, USA; 6 Center for Reproductive Medicine Renji Hospital, School of Medicine, Shanghai Jiao Tong University, Shanghai 200025, China; 7 Nezhat Medical Center, Atlanta Center for Special Minimally Invasive Surgery and Reproductive Medicine, Atlanta, GA 30342, USA

**Keywords:** endometriosis, chronic pelvic pain, unexplained infertility, free Endometriosis Risk Advisor mobile application, laparoscopic surgery, robotic surgery

## Abstract

Endometriosis is a prevalent condition that affects millions of individuals globally, leading to various symptoms and significant disruptions to their quality of life. However, the diagnosis of endometriosis often encounters delays, emphasizing the pressing need for non-invasive screening. This retrospective cross-sectional study aimed to evaluate the utility of the Endometriosis Risk Advisor (EndoRA) mobile application in screening for endometriosis in patients with chronic pelvic pain and/or unexplained infertility. The study consisted of 293 patients who met specific criteria: they were English-speaking individuals with chronic pelvic pain and/or unexplained infertility, owned smartphones, and had no prior diagnosis of endometriosis. The results demonstrated that the EndoRA score exhibited a high sensitivity of 93.1% but a low specificity of 5.9% in detecting endometriosis. The positive predictive value was 94.1%, while the negative predictive value was 5.0%. Although the study had limitations and potential selection bias, its findings suggest that EndoRA can serve as a valuable screening tool for high-risk individuals, enabling them to identify themselves as being at an increased risk for endometriosis. EndoRA’s non-invasive nature, free access, and easy accessibility have the potential to streamline evaluation and treatment processes, thereby empowering individuals to seek timely care and ultimately improving patient outcomes and overall well-being.

## 1. Introduction

Endometriosis is a systemic, inflammatory, estrogen-dependent condition characterized by endometrial stroma and gland-like lesions outside of the uterus [1,2]. The lesions may present as superficial peritoneal, ovarian endometrioma, and/or deep infiltrating endometriosis [3]. Clinical symptoms range from asymptomatic to dysmenorrhea, dyschezia, dyspareunia, organ dysfunction, catamenial pneumothorax, and/or infertility [4,5,6,7].

Endometriosis affects 190 to 700 million patients, including up to 80% with pelvic pain and up to 47% seeking fertility treatment [8,9,10,11]. Endometriosis is concomitantly present in up to 87% of patients with symptomatic uterine leiomyomas, with approximately 700 million patients having leiomyomas during their reproductive years [11,12,13]. Although pain and infertility are common concerns for patients with endometriosis, the real toll is even greater: patients diminished quality of life, increased incidence of depression, adverse effects on intimate relationships, higher risk of cancer, higher healthcare costs, indirect impact on the economy through work absenteeism, and short-term and long-term disability [14,15,16,17,18,19,20].

Despite these adverse sequelae of endometriosis, the average delay from symptom onset to surgical diagnosis, which remains the gold standard, is 4–11 years [20,21,22,23]. It has been estimated that 47% of patients with endometriosis had been seen at least five times by a doctor prior to an endometriosis diagnosis or referral for their symptoms [8]. Varying presentations, limited access to endometriosis specialists, and reserving surgery as a last resort, especially in adolescents, may contribute to diagnosis delay [24,25,26,27]. Furthermore, with improved outcomes in Assisted Reproductive Technology (ART), many reproductive endocrinology and infertility specialists are increasingly in favor of clinical diagnosis and medical management versus surgical diagnosis and excision before treatment for unexplained infertility [28,29].

As minimally invasive surgery is not always accessible, the search for a non-invasive test to screen for patients with endometriosis is needed [23,30]. Machine learning algorithms, patient questionnaires, imaging techniques, and biomarkers (e.g., BCL6) have been proposed for endometriosis screening [23,30,31,32,33,34,35,36].

The free Endometriosis Risk Advisor (EndoRA) mobile application (https://endometriosis.app) (accessed on 30 June 2023) has been developed by an artificial intelligence (AI)-based algorithm by the philanthropic organization Worldwide Endomarch (Endomarch)^®^ and donated to patients at risk for endometriosis as a mobile application using a series of questions that aim to calculate the risk of endometriosis (low: <50%; moderate: 50–75%; and high: >90%) [37,38].

The development of EndoRA underwent a meticulous and comprehensive process, beginning with the design of questions by a team of highly experienced endometriosis specialists. Expert evaluation and validation followed, encompassing both questions and answers, with valuable contributions from engineers and AI specialists, resulting in an application of great reliability and accuracy [38]. Validation procedures involved the use of a neural network and diverse patient datasets to establish performance metrics, prevent overfitting, and validate hyperparameters during the training process. After several improvements and modifications, EndoRA was launched in October 2019 and has been downloaded over 10,000 times. This free mobile application holds immense value as a screening tool, empowering high-risk patients to self-identify their elevated risk for endometriosis. Our report highlights its utility as an accessible and convenient screening tool for the presence of endometriosis, potentially identifying patients with a heightened index of suspicion and expediting the evaluation and treatment process. 

## 2. Materials and Methods

This is a retrospective cross-sectional study from September 2019 to March 2022. The study was approved by the West Coast IRB committee.

### 2.1. Subjects

In this study, we conducted a review of medical records for a total of 558 patients. Among these individuals, we identified 293 patients who met our inclusion criteria and were actively seeking medical care for chronic pelvic pain and/or unexplained infertility. The study focused specifically on patients between the ages of 17 and 49 who were referred for surgical evaluation of chronic pelvic pain and/or unexplained infertility. The inclusion criteria encompassed English-speaking individuals with chronic pelvic pain and/or unexplained infertility who had access to smartphones and had not received a prior diagnosis of endometriosis. These patients underwent video-laparoscopic surgery, either with or without robotic assistance. The exclusion criteria consisted of patients who had previously been diagnosed with endometriosis through surgical and/or histopathologic means, as well as those who were unable or unwilling to use the mobile application. Prior to their initial consultation, eligible patients were asked to download the Endometriosis Risk Advisor mobile application and report their scores. Comprehensive medical histories and thorough gynecologic physical examinations were obtained from clinical visits for all patients, and the relevant data were extracted for analysis. In this study, pelvic pain was defined as pain experienced in the lower abdominal and pelvic regions, which encompassed symptoms like dysmenorrhea, dyspareunia, dyschesia, and chronic pelvic pain. Infertility refers to the inability to conceive after 12 or more cycles of unprotected intercourse for individuals under 35 or after 6 cycles for those over 35 [39,40].

### 2.2. Surgical Evaluation

All surgical procedures were performed by a highly skilled minimally invasive gynecological surgeon who possessed expertise in treating endometriosis. The surgeon was assisted by one or two fellows specializing in minimally invasive gynecology.

The patients consented to a combined procedure of video-laparoscopy (Stryker) with or without robotic assistance (Intuitive) and video hysteroscopy (Stryker) to evaluate the abdominal, pelvic, and uterine cavities, as well as to assess tubal patency. Diagnostic hysteroscopy was conducted by instilling normal saline into the uterine cavity and evaluating it using a high-quality Stryker 2.9-mm rigid optic with a 3.7-mm sheath (Portage, MI, USA) [2]. Following the removal of the hysteroscope, a HUMI uterine manipulator (Trumbull, CT, USA) was inserted into the uterine cavity.

**Figure 1:**
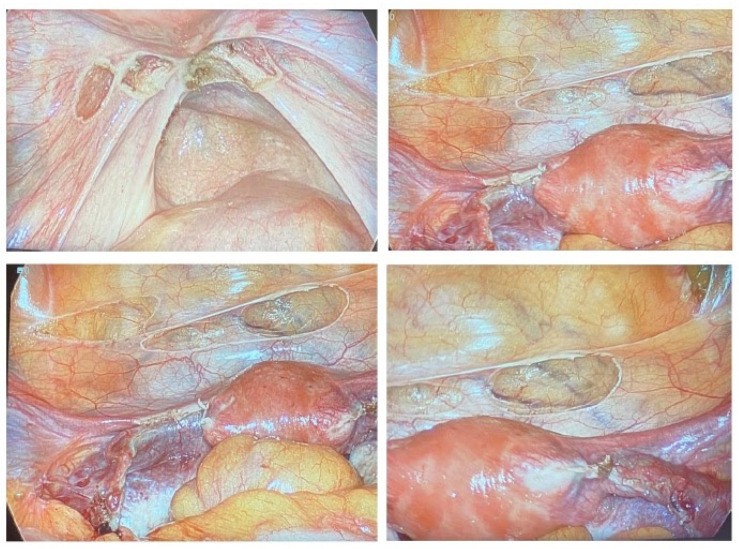
This patient presented with a primary complaint of pelvic pain. Video laparoscopic findings did not reveal any obvious evidence of endometriosis. To rule out endometriosis and decrease the chance of missing endometriosis, as demonstrated above, multiple biopsies were taken from the most common sites of endometriosis away from tubes and ovaries.

The abdominal cavity was entered via a video-laparoscope by creating pneumoperitoneum using a Verres needle under a low pressure of 0–10 mm of mercury [2]. Next, we inserted one 5–10 mm umbilical port and one to three 5 mm lower abdominal wall ports, depending on the extent of the pathology found [2]. The entire abdominal and pelvic cavities were thoroughly evaluated for any obvious or suspicious lesion(s) related to endometriosis, deep infiltrative fibrosis, or adhesions secondary to endometriosis. Special attention was given to the peritoneal and omental surfaces, bilateral hemidiaphragm, liver, gallbladder, stomach, small and large intestines, uterus, bilateral adnexa, and pelvic sidewalls [2]. Biopsies and resections for abdominal and pelvic pathology were collected for histopathological evaluation using cold scissors, radiofrequency, or CO_2_ laser techniques. In cases of minimal stage one endometriosis and cases with no obvious endometriosis and/or suspicious lesions, multiple random biopsies were taken, as demonstrated in Figure 1. These biopsies included three from the anterior cul-de-sac, three from the posterior cul-de-sac, one or two from the right pelvic and abdominal side wall, and one or two from the left pelvic and abdominal side wall. All collected tissue samples were properly labeled according to the corresponding anatomical structures and sent to the Department of Pathology at Stanford University Medical Center for histological evaluation. The lesions were classified in the operating room as follows: Group one included obvious, typical endometriosis lesions. Group two comprised atypical endometriosis lesions, such as white, blue, or purple star-like lesions. Group three consisted of fibrotic or adhesive lesions in patients without prior abdominal surgeries, which included all the patients in this cohort. For the purpose of classification, there is also a group four that includes patients with fibrosis or adhesions resulting from prior abdominal surgery. However, none of the patients in this cohort had undergone previous abdominal surgery, so there were no group four samples sent to the pathology lab for these patients. During the video-laparoscopy, normal saline or dye was injected through the HUMI uterine manipulator to assess the fallopian tubes for tubal patency [2]. For patients with macroscopic endometriosis, clinical staging was performed using the rASRM scoring system [41]. The patients are classified into four groups (I–IV) based on the severity of endometriosis, with Group IV representing the most severe cases. Surgical records and histopathology reports were collected for data extraction. The study evaluated the screening utility of the EndoRA mobile application by comparing it to the gold standard of histopathology for diagnosing endometriosis in patients with chronic pelvic pain and/or unexplained infertility.

### 2.3. The Endometriosis Risk Advisor

The free Endometriosis Risk Advisor (EndoRA) is developed by an AI-based algorithm that categorizes patients based on their chief complaint: infertility or pain. A series of questions follows, focusing on symptomatology, family history, psychiatric history, past medical history, fertility issues, and prior fertility testing. These questions are designed to gather additional information based on the patients’ previous answers, with the questionnaire dynamically adapting based on their responses. The AI-based algorithm then calculates the risk assessment as low risk (<50%), moderate risk (50–75%), or high risk (>90%) of possibly having endometriosis (https://endometriosis.app) (accessed on 30 June 2023).

EndoRA’s development has undergone a thorough and comprehensive process. In the initial phase, all the questions were designed by the senior author and his team, with a combined experience of over 100 years in treating patients with endometriosis. Subsequently, a group of endometriosis experts evaluated and validated these questions. Following that, both the questions and answers underwent evaluation and validation by a team of experienced engineers, AI experts, and specialists to develop the application. It is important to note that the EndoRA application has undergone validation to ensure its reliability and accuracy [38]. The validation process encompassed medical literature up to the end of 2018, along with other data, including inputs from endometriosis experts. The aim was to utilize all available data to enhance the app’s accuracy. This comprehensive approach involved collaboration with endometriosis experts, fellows, students, and individuals acknowledged in the acknowledgment section for their valuable contributions to the validation and revision process. Patient data played a crucial role in shaping the app’s development as well.

The application validation process involved a neural network that collected, tested, and evaluated a diverse and representative dataset of individual patients. Performance metrics were defined as follows: high accuracy, 90 percent or more; average accuracy, 50 to 75 percent; and low accuracy, less than 50 percent. Subsequently, the dataset was split into training, validation, and testing sets to train the AI model, validate hyperparameters, prevent overfitting, and evaluate the final performance. K-fold cross-validation was employed. The app’s performance was compared with the expertise of highly experienced specialists in endometriosis, reproductive endocrinology, and infertility. It was evaluated for errors and potential mistakes, leading to several improvements and three modifications before its final launch in October 2019. Since then, the application has been downloaded more than 10,000 times, and all the feedback received has been positive. The app has been thoroughly checked numerous times to ensure fairness and avoid any bias or discrimination. Additionally, compliance with security and privacy measures has been achieved to protect user data. Continuous collaboration with the experts who developed the app is maintained to facilitate updates and ongoing improvements.

### 2.4. Statistical Analysis

STATA Version 17 was utilized to conduct all statistical analyses. The results were presented as numbers, percentages, and mean ± SD for quantitative and qualitative variables. Cross-tabulations were employed to determine the sensitivity, specificity, positive predictive value, and negative predictive value of EndoRA scores, with surgical histopathological tissue diagnosis serving as the gold standard. Additionally, likelihood ratios (LR) were used to calculate the probability of disease while adapting to varying prior probabilities of disease in different contexts. The positive likelihood ratio was calculated as sensitivity divided by 1-specificity, while the negative likelihood ratio was calculated as 1-sensitivity divided by specificity. The diagnostic odds ratio (DOR) was also calculated using the following formula: (True Positive/False Negative)/(False Positive/True Negative). Accuracy was also calculated as (True Positive + True Negative)/(True Positive + False Positive + True Negative + False Negative).

## 3. Results

The analysis was performed on complete data from 293 patients who were referred to our practice for surgical evaluation of chronic pelvic pain and/or unexplained infertility and met the inclusion criteria of our study. The patients had an average age of 35.79 ± 0.4 years (range 17–49) and an average body mass index (BMI) of 24.16 ± 0.3 kg/cm^2^ (range 15.8–44.6). Surgical histopathology confirmed endometriosis in 94.2% (*n* = 276) of the patients showing endometrial-like stroma and glands in their pathology report. In confirmed cases of endometriosis, the prevalence of endometriosis in the patient population varied across different surgical stages, with 12.7%, 34.4%, 16.7%, and 36.2% for Stages I, II, III, and IV, respectively. Table 1 provides detailed information on the baseline characteristics of the study population.

In this evaluation, a high-risk EndoRA score was considered positive, while a moderate or low EndoRA score was considered negative. The sensitivity and specificity of the EndoRA score were assessed in comparison to the gold standard test of surgical histopathology. The sensitivity was calculated to be 93.1%, representing the proportion of true positive cases identified by the EndoRA score. The specificity was calculated to be 5.9%, representing the proportion of true negative cases identified by the EndoRA score. The positive predictive value (PPV) of the EndoRA score was calculated to be 94.1%. This indicates the percentage of patients with high-risk EndoRA scores who were confirmed to have positive endometriosis based on surgical histopathology. The negative predictive value (NPV) of the EndoRA score was calculated to be 5.0%, representing the percentage of patients with low or moderate-risk EndoRA scores who were confirmed to be negative for endometriosis in surgical histopathology. The screening performance of the EndoRA score in detecting endometriosis as compared to surgical histopathology was also analyzed in different groups, including those with infertility, pain, and various stages of endometriosis. Table 2 provides detailed values for the EndoRA score in each group.

## 4. Discussion

Given the profound impact of endometriosis on both patients’ lives and society as a whole, it is crucial to minimize the time between the onset of symptoms and diagnosis. This can be achieved by improving patient education and ensuring timely referral to endometriosis specialists [1,16]. However, the evaluation for endometriosis is only the initial step. For many patients who desire future fertility or struggle with conception, as well as those experiencing chronic pain, treatment may be necessary. By increasing awareness and suspicion of endometriosis, physicians can expedite referrals to fertility specialists or endometriosis specialists, consider oocyte cryopreservation at an earlier age for individuals at higher risk of endometriosis, identify patients at greater risk for specific subtypes of ovarian cancer, and provide tailored surveillance and preventive interventions [42]. When encountering patients with chronic pelvic pain and/or unexplained infertility, utilizing the free Endometriosis Risk Advisor (EndoRA) mobile application can alert healthcare providers to the potential presence of endometriosis and streamline the evaluation and treatment process. 

The study aimed to evaluate the utility of the EndoRA as a non-invasive screening test for endometriosis in a high-risk population, specifically among patients experiencing chronic pelvic pain and/or unexplained infertility. Though the EndoRA’s specificity is low (5.9%), the high sensitivity of 93.1% will result in fewer cases of endometriosis being missed. In our study, the EndoRA has a high PPV (94.1%) and a low NPV (5%). This reflects the higher prevalence of endometriosis diagnosed in our referral center. In particular, for patients with unexplained infertility, the EndoRA has a high sensitivity (94.0% for patients with infertility and 95.1% for patients with infertility and pain). In this case, utilizing a mobile application for endometriosis screening offers distinct advantages over an endometrial biopsy. The application allows for self-directed screening, is free, carries no procedural risks or discomfort, and provides instant results, making it particularly beneficial for high-risk groups.

While our study demonstrated high sensitivity and positive predictive value for EndoRA in detecting endometriosis, it is important to acknowledge the limitations that may have influenced our results. One significant limitation is the presence of selection bias, as the patients included in our study were already referred to our practice for surgical evaluation, indicating a pre-existing consideration for surgery due to suspicion for endometriosis. This bias may have affected the distribution of EndoRA scores, leading to a scarcity of low-risk EndoRA scores in our study. Additionally, the high prevalence of unsuccessful medical management of endometriosis among our patients could have influenced the predictive values of EndoRA. It is essential to highlight that the EndoRA mobile application is specifically designed to be useful for high-risk groups rather than the general population.

Furthermore, our findings indicate that EndoRA exhibits a higher screening value in patients with stages III and IV endometriosis, as it was able to screen these cases with a higher degree of accuracy (sensitivity: 96.8%, specificity: 100%, PPV: 100%) versus patients with stages I and II endometriosis. However, further research is needed to confirm the utility of EndoRA in broader populations at higher risk for endometriosis. Moving forward, it is crucial to refine the screening questions within the application to enhance its ability to identify early stages of endometriosis for earlier treatment consideration. Additionally, exploring the use of EndoRA in practices with a lower prevalence of endometriosis will provide valuable insights into its utility in different patient populations.

The current study focused on a specific group of patients at high risk of endometriosis, limiting the generalizability of the findings to a broader population. However, recognizing the importance of broader applicability, future research should explore EndoRA’s effectiveness in a general population, including teenagers. By extending investigations to a wider demographic, valuable insights into EndoRA’s implications and benefits can be gained. Additionally, examining its impact on reducing diagnostic delays and providing timely medical support for individuals beyond the high-risk group presents a promising avenue for further research. Such efforts could significantly contribute to improving healthcare outcomes for a diverse range of individuals.

For future research, there are several potential avenues to enhance EndoRA’s specificity. Firstly, fine-tuning the algorithms and machine learning models can be explored as technology advances and additional relevant medical and non-medical data becomes available. Regularly incorporating and updating such data while optimizing the features used for prediction can further enhance EndoRA’s specificity and accuracy. Continuous efforts toward refining the models and incorporating the latest advancements in the field will be crucial to achieving better results. Secondly, the consideration of incorporating biomarkers or other diagnostic indicators may help strengthen the accuracy of the predictions. Lastly, conducting further validation studies with larger and more diverse patient populations would also be beneficial in refining EndoRA’s specificity and ensuring its effectiveness across different patient profiles.

Patient education, timely referral, and a shift in physician approach are essential elements for improving the care of patients with endometriosis [18]. To potentially contribute to the standardization and democratization of care for patients with endometriosis, the free Endometriosis Risk Advisor (EndoRA) mobile application was launched in October 2019. Since its launch, approximately 10,000 people have downloaded the application. The Worldwide Endomarch (Endomarch^®^) organization has donated this application to patients with chronic pelvic pain and/or unexplained infertility who may have endometriosis, and some of them may not have the means for surgical and non-surgical diagnostic tests or procedures [37].

The EndoRA application is readily available on iOS and Android smartphones, offering convenience and accessibility to users. Unlike invasive procedures such as endometrial biopsies or blood draws, EndoRA does not pose any procedural risks. Moreover, it eliminates the need for a doctor’s visit for administration, as is the case with imaging evaluations. This aspect is particularly advantageous for regions where healthcare services, especially specialized endometriosis specialists and diagnostic and operative video-laparoscopy, are not easily accessible.

By empowering high-risk patients, the EndoRA mobile application may serve as a valuable screening tool, allowing individuals to recognize their elevated risk for endometriosis. It offers a non-invasive, user-friendly solution that may aid in early detection and prompt further evaluation and treatment in high-risk groups. This free application may be a valuable resource in guiding high-risk patients with chronic pain and/or infertility, especially those with limited financial resources, to seek appropriate help and medical attention.

The availability of the free EndoRA application, its provision of instant feedback, and the absence of procedural risks may encourage patients at higher risk of endometriosis to take a proactive approach to seeking professional evaluation sooner. It may also prompt these patients with a higher risk of endometriosis to consider oocyte cryopreservation or other measures to address this condition at a younger age. The high sensitivity of EndoRA can be particularly valuable for clinicians to identify potential endometriosis in patients with chronic pelvic pain and/or unexplained infertility. This, in turn, can expedite referral to proper specialists for accurate diagnosis and appropriate treatment. For patients with unexplained infertility and unsuccessful in vitro fertilization (IVF) attempts or other measures, including but not limited to hormonal suppression, surgical evaluation and treatment of endometriosis may be an option and may provide additional benefits within this subgroup of patients [43].

## Figures and Tables

**Table 1:** Demographic characteristics of study population.

Variable	Number (%)/Mean ± SD
Age (year)	35.79 ± 0.4
BMI (kg/cm^2^)	24.16 ± 0.3
Nulligravida	169 (59.1)
Smoking (current or past)	18 (6.2)
Family History of Endometriosis	41 (14.0)
Pelvic Pain	233 (81.5)
Unexplained Infertility	212 (75)
Pelvic Pain and Unexplained Infertility	148 (50.5)
Endometriosis Stage (rASRM)	
I	35 (12.7)
II	95 (34.4)
III	46 (16.7)
IV	100 (36.2)

BMI—Body Mass Index (weight in kg/height in cm^2^).

**Table 2:** Screening performance of the Endometriosis Risk Advisor (EndoRA) in comparison to the gold standard, histopathology.

	Total	Infertility Group	Pain Group	Infertility and Pain Group	Stage I/II	Stage III/IV
Sensitivity (%)	93.1	94.0	93.5	95.1	88.3	96.8
Specificity (%)	5.9	9.1	N/A	N/A	N/A	100.0
PPV (%)	94.1	95.0	94.5	95.8	86.9	100.0
NPV (%)	5.0	7.7	N/A	N/A	N/A	16.7
PLR	0.98	1.03	0.93	0.95	0.88	N/A
NLR	1.17	0.65	N/A	N/A	N/A	0.03
DOR	0.84	1.57	N/A	N/A	N/A	N/A
Accuracy %	88.1	89.6	88.8	91.2	77.9	89.6

PPV—Positive Predictive Value; NPV—Negative Predictive Value; PLR—Positive Likelihood Ratio; NLR—Negative Likelihood Ratio; DOR—Diagnostic Odds Ratio; N/A—could not be calculated with our data.

## Data Availability

Data will be made available to the editors of the journal for review or query upon request.
